# Oophorectomy Reduces Estradiol Levels and Long-Term Spontaneous Neurovascular Recovery in a Female Rat Model of Focal Ischemic Stroke

**DOI:** 10.3389/fnmol.2018.00338

**Published:** 2018-09-13

**Authors:** Paolo Bazzigaluppi, Conner Adams, Margaret M. Koletar, Adrienne Dorr, Aleksandra Pikula, Peter L. Carlen, Bojana Stefanovic

**Affiliations:** ^1^Sunnybrook Research Institute, Sunnybrook Health Sciences Centre, Toronto, ON, Canada; ^2^Adult Vascular Neurology, Toronto Western Hospital, Toronto, ON, Canada; ^3^Fundamental Neurobiology, Krembil Research Institute, Toronto, ON, Canada; ^4^Department of Medical Biophysics, University of Toronto, Toronto, ON, Canada

**Keywords:** arterial spin labeling, estrogen, ischemia, ovariectomy, local field potential

## Abstract

Although epidemiological evidence suggests significant sex and gender-based differences in stroke risk and recovery, females have been widely under-represented in preclinical stroke research. The neurovascular sequelae of brain ischemia in females, in particular, are largely uncertain. We set out to address this gap by a multimodal *in vivo* study of neurovascular recovery from endothelin-1 model of cortical focal-stroke in sham vs. ovariectomized female rats. Three weeks post ischemic insult, sham operated females recapitulated the phenotype previously reported in male rats in this model, of normalized resting perfusion but sustained peri-lesional cerebrovascular hyperreactivity. In contrast, ovariectomized (Ovx) females showed reduced peri-lesional resting blood flow, and elevated cerebrovascular responsivity to hypercapnia in the peri-lesional and contra-lateral cortices. Electrophysiological recordings showed an attenuation of theta to low-gamma phase-amplitude coupling in the peri-lesional tissue of Ovx animals, despite relative preservation of neuronal power. Further, this chronic stage neuronal network dysfunction was inversely correlated with serum estradiol concentration. Our pioneering data demonstrate dramatic differences in spontaneous recovery in the neurovascular unit between Ovx and Sham females in the chronic stage of stroke, underscoring the importance of considering hormonal-dependent aspects of the ischemic sequelae in the development of novel therapeutic approaches and patient recruitment in clinical trials.

## Introduction

Epidemiological evidence suggests lower risk of stroke among *premenopausal* women than among age-matched men (Stegmayr et al., 1997; Sudlow and Warlow, 1997). By the *perimenopausal* years (55- to 64-year olds), the stroke risk for women equalizes to that of age-matched men (Anderson et al., 1991). Once in *post-menopausal* stage, women who experienced stroke show less post-stroke recovery than men: they are more likely than men to be institutionalized, and they tend to suffer worse post-stroke disability (Bushnell, 2008; Petrea et al., 2009). The hormonal profile progression thus plays a major role in determining women’s susceptibility and vulnerability to stroke. A recent report (Levine et al., 2016) suggested that bilateral oophorectomy accelerates aging (particularly of the circulatory system), as measured by increased methylation. Patient studies (Ingelsson et al., 2016; Levine et al., 2016; Rocca et al., 2016, 2017) showed an association between pre-menopausal bilateral oophorectomy and the accumulation rate of cardiovascular diseases and multimorbidity of 18 defined chronic conditions. In the United States, an estimated 2 million women have undergone surgically induced menopause from hysterectomy with removal of the ovaries between 2000 and 2004 alone (Diaz Brinton, 2012), and a more recent survey suggests just below 500,000 new hysterectomies in 2009 (Cohen et al., 2014). Importantly, women undergoing oophorectomy (i.e., surgical menopause) show lower levels of estrogen than age-matched women experiencing natural menopause (Korse et al., 2009). While the epidemiological evidence thus clearly shows that sex and estrogen levels are important factors in the risk of stroke and in its long-term outcome, the mechanism of these dependencies is unclear. In light of the above-mentioned complexity and the slow evolution of cerebrovascular impairments that are thought at the heart of this interaction, examining the mechanism of sex-based differences in stroke risk and its outcomes is exceedingly difficult in humans (Rocca et al., 2017). Preclinical models are hence key as they allow detailed investigation of neurovascular changes in well-controlled conditions. To date, however, estradiol’s roles have been investigated almost exclusively *in vitro* (McCullough and Hurn, 2003). With respect to age, experimental stroke has been shown to increase edema and result in larger strokes in middle-aged female animals compared with younger females with intact gonadal function [for review (Manwani and McCullough, 2011)]. However, neurovascular functioning in the chronic stage post ischemia in females, and its dependence on ovariectomy and estrogen decline, has not been evaluated.

In the present work, we modeled the estrogen drop observed in pre-menopausal women following oophorectomy and determined the effects of ovariectomy on spontaneous neurovascular recovery in the chronic stage post focal cortical ischemia. To induce a drop in estrogen levels, we used 7–9 months old retired-breeder female rats [known to best resemble the physiological changes of women in their fourth decade (Sengupta, 2013)], which underwent ovariectomy (Ovx) or sham surgery. Two weeks thereafter, focal cortical stroke was induced by microinjection of Endothelin-1 (ET-1), (Lake et al., 2017) and serum estrogen levels were measured instead of estrous cycle monitoring (Singletary et al., 2005; Weixelbaumer et al., 2014). In our previous report using the same model in the male population (Lake et al., 2017), we reported resting perfusion normalization and sustained cerebrovascular hyperreactivity, associated with increased neuronal excitability, 3 weeks after stroke. In light of the functional nature of these readouts that cannot be captured by post-mortem assays and the fact that T_2_-weighted MRI sensitivity parallels histopathology (i.e., cresyl-violet) in detecting stroke volume in the focal cortical ET-1 model (Biernaskie et al., 2001), we used quantitative MRI of cerebral blood flow and intracerebral multielectrode electrophysiological recordings to compare stroke size and neurovascular state in the peri- and contra-lesional cortex 3 weeks following stroke induction. Topographical analysis of local field potentials revealed neuronal deficits within 0.5 mm of the ET-1 injection site in the Ovx group, which were inversely correlated to the level of estradiol at the time of stroke. Despite comparable stroke volumes of the two cohorts, the Ovx females displayed reduced peri-lesional blood flow and widespread exaggerated cerebrovascular responses to hypercapnia when compared to Sham animals. Low estrogen levels were thus shown to be associated with exacerbated long-term neurovascular damage from ischemic stroke.

## Materials and Methods

All experimental procedures in this study followed the ARRIVE guidelines and were approved by the Animal Care Committee of the Sunnybrook Research Institute, which adheres to the Policies and Guidelines of the Canadian Council on Animal Care and meets all the requirements of the Provincial Statute of Ontario, Animals for Research Act as well as those of the Canadian Federal Health of Animals Act. Animals were housed in pairs on a standard 12-h light/dark cycle. Food and water were freely available. Twenty-five mature female Sprague Dawley rats were purchased from Charles River Canada. They were retired breeders (7–9 months old at the beginning of the study and having had at least one litter), with mean weight at the beginning of the study of 420 ± 20 g. Animals were randomly assigned to Ovx or Sham groups and the researcher performing MRI and electrophysiology experiment and data analysis was blinded to the surgery level of the animal. Following earlier work (Bernal-Mondragón et al., 2013), we let animals recover from Ovx surgery for 2 weeks before stroke induction surgery. This period was necessary to minimize mortality and to allow a measurable drop in estrogen serum levels to develop [see results, (Laughlin et al., 2000; Strom et al., 2008, 2010)]. In line with 3R principles and to allow recovery from the imaging experiments, we avoided the use of alpha-chloralose anesthesia, which although frequently employed in non-survival studies is associated with prolonged and poor recovery (Silverman and Muir, 1993; Flecknell, 2016). Instead, Propofol anesthesia (see details below) was used throughout all imaging and electrophysiological recordings.

### Ovariectomy

Given the expected high mortality of the Ovx procedure, sixteen females were randomly assigned to the Ovx group and eight to the Sham group. Bilateral Ovx was conducted using strict sterile surgical techniques. Rats were anesthetised with isoflurane (5% induction, 2.5% maintenance). A blood sample for serum estradiol measurement was acquired from the lateral tail vein. The bilateral Ovx followed a 2 cm incision being made on each lateral side of the abdomen (between the last rib and hip). The ovary and adjacent uterine horn was isolated and clamped. The ovary and fallopian tube was ligated with 4-0 silk suture and then excised. The abdomen was sutured closed. All rats received subcutaneous local anesthetic (bupivicaine HCl 7 mg/kg, Marcaine, Hospira Healthcare Corp.) along the incision site, analgesic (buprenorphine 0.1 mg/kg, Temgesic, Reckitt Benckiser Healthcare), and antibiotics (enrofloxacin 5 mg/kg, Baytril, Bayer Inc.). Sham surgeries were conducted exactly in the same way, except that the ovary and uterine horn remained intact. Following surgery, animals were returned to the colony for a 2 weeks recovery period. Six rats died during the first 24 h after the Ovx surgery (three animals chewed the stiches reopening the wound and were hence excluded from the study, and other three died on unknown reasons).

### Stroke Induction

Ovx and Sham animals underwent the stroke induction procedure, 2 weeks after surgery, under isoflurane anesthesia (5% induction and 2–2.5% maintenance) following the procedures we described in Lake et al. (2017). In brief, animals were secured in a small animal stereotaxic apparatus (David KOPF Instruments, Tujunga, Los Angeles, CA, United States). A blood sample for serum collection was acquired from the lateral tail vein. Under aseptic condition, two burr holes were drilled over the right sensorimotor cortex (AP +2.3 mm and AP -0.5 mm; ML 2.1 mm) using a high-speed micro-drill (Foredom Electric Co., Bethel Connecticut, United States). A 10-μl Hamilton Syringe (Model 80366, 26-gauge needle with beveled tip) was used to inject 800 pmol of ET-1 (Calbiochem, Millipore, Billerica, MA, United States, dissolved in sterile saline) at -2.3 mm DV (dorsal-ventral) in both locations. One 2-μl aliquot was injected at each location, for a total of 4 μl. The scalp was sutured over the skull. Four Ovx females and two Sham females did not survive stroke induction surgery, leaving six animals in each cohort.

### Magnetic Resonance Imaging

The current study was conducted in a 7T horizontal pre-clinical MRI scanner from Bruker BioSpec. The rats were anesthetized with isoflurane, intubated using 14-gauge i.v. catheters, and mechanically ventilated using a rodent ventilator (SAR-830/P, CWE Inc.). Isoflurane was discontinued and intravenous propofol (PharmaScience Inc.) infusion commenced, at a rate of 43 mg/kg/hour. Physiological status was monitored by pulse oximetry (MouseOx Plus MRI compatible, STARR Life Sciences Corp.) and end-tidal capnography (microCapStar, CWE Inc.), with core body temperature maintained using a rectal probe feedback controlled water bed (1025T, Small Animal Instruments Inc.).

### T_2_-Weighted MRI

A birdcage body coil was used for signal excitation and a quadrature receive-only coil for signal detection. Forty-five coronal images were obtained with a rapid acquisition with relaxation enhancement (RARE) sequence (RARE factor of eight, repetition time/echo time TR/TE of 5500/47 ms, and a matrix size of 128 × 256, flip angle 180), with a nominal in-plane spatial resolution of 0.1 mm × 0.1 mm and a slice thickness of 0.5 mm in under 12 min. Images were imported into Display (from the MNI Minc package developed at the Brain Imaging Centre of the Montreal Neurological Institute) for semi-automated segmentation. Following earlier work (Neumann-Haefelin et al., 2000; Kidwell et al., 2003; van der Zijden et al., 2008; Lake et al., 2017), stroke regions were segmented using a predetermined signal intensity threshold of greater than two standard deviations (SDs) above the mean contra-lesional cortical signal intensity. Following segmentation, stroke volumes were calculated for each animal.

### CASL Data Acquisition

For CASL imaging, a custom built labeling coil was placed at the level of the common carotid arteries. The position of the labeling coil relative to the imaging coil was then determined with a localizer acquired using the labeling coil (i.e., Tripilot sequence) to ensure optimal labeling offset in all subjects (**Supplementary Figure S1A**). Shimming was next performed using Fastmap sequence (Bruker) to adjust 1st and 2nd order shims in the brain region to be imaged (containing the stroke volume and relevant brain landmarks of interest, **Supplementary Figure S1B**). The resulting shim was assessed using a PRESS sequence (TE/TR 20/2500 ms, 1 average, 2048 points, 13 ppm spectral width) to measure FWHM of the free induction decay of water. Finally, we collected a 2-min series of CASL-EPI images and evaluated the difference between the control and labeled frames to ensure adequate labeling efficiency. We then proceeded with the collection of the full CASL protocol. Using a 1.5-s adiabatic labeling pulse and a 0.4-s post-labeling delay, single average, single shot continuous arterial spin labeling (CASL) echo planar images (EPI) were obtained with a 0.25 mm × 0.25 mm in-plane resolution, and TR/TE of 2500/9.2 ms, matrix size 80 × 57, 1.4 Partial-FT Acceleration, FOV 20 mm × 20 mm, effective spectral bandwidth 250 MHz (Lake et al., 2017). EPI readout details are reported in the **Supplementary Information**. A single 1.5-mm thick coronal slice was positioned 1 to 2 mm caudally to the caudal ET-1 injection site. CASL was performed during medical air and hypercapnic mixture breathing to measure cerebrovascular reactivity to 10% carbon dioxide, a standard test of cerebrovascular function employed in both preclinical and clinical research. For CO_2_ challenges, the composition of inhalant gasses was controlled by programming a gas mixer (GSM-3, CWE Inc.). An initial baseline (4 min of 30% O_2_, 70% N_2_) was followed by four hypercapnic challenges (1 min each of 10% CO_2_, 30% O_2_, and 60% N_2_) alternating with normocapnic periods (4 min each of 30% O_2_, 70% N_2_). Oxygen enrichment (30% vs. 21%) was used to compensate for respiratory depression induced by anesthesia. At the end of the MRI experiment, the propofol infusion was discontinued and the animal recovered to colony for 3–4 days, in preparation for electrophysiological recordings.

### CASL Data Processing

For analysis of functional MRI experiments, we used Analysis of Functional NeuroImages (AFNI, NIH); CASL data were analyzed as in our previous studies (Lake et al., 2015, 2016, 2017): motion was corrected using 2dImReg; Gaussian blurring of masked gray matter was performed using 3dBlurToFWHM (with full-width half max of 0.55 mm); and generalized linear modeling done using 3dDeconvolve (with FDR correction). Subject-specific hemodynamic response functions were produced by averaging the signal in the left (contra-lateral) cortical gray matter (Kang et al., 2003). A threshold was applied to maps of perfusion signal changes elicited by hypercapnia and resting perfusion to correct for multiple comparisons (false discovery rate *q* < 0.01). In each animal, peri-lesional ROI and the homologous region in the contralateral hemisphere used as the within-subject reference ROI, were delineated using anatomical local contrast on the EPI and T_2_-weighted structural scans (**Figure 3Ai**). Following earlier work, voxels with contrast’s effect size exceeding 2 Median Absolute Deviations from contrast’s median (estimated independently per surgery group, ROI, and contrast) were excluded from further analysis. Resting perfusion was expressed in absolute units (ml/100 g tissue/min) based on the model of ASL signal described by Hendrich et al. (2001), while assuming a lambda (blood–brain partition coefficient) of 0.9 ml/g (Roberts et al., 1996), longitudinal relaxation time of gray matter at 7T of 1.6 s for neocortex (Guilfoyle et al., 2003), and inversion efficiency of 0.7 following our prior work in this model (Gomez-Smith et al., 2017).

### Electrophysiological Data Acquisition

The rats were anesthetized with isoflurane and positioned inside the stereotaxic apparatus. The lateral tail vein was cannulated with a 24-gauge intravenous catheter. Two bilateral craniotomies were created around the ET-1 stroke injection sites from AP +2.3 mm to -4.0 mm and ML ±0.5 mm to ±5.0 mm. After removal of the skull bone, the *dura mater* was excised and the cortical surface kept hydrated with sterile phosphate buffered saline. The continuous infusion of propofol was then commenced at a rate of 43 mg/kg b.w./hour. Physiological status was monitored by pulse oximetry (MouseOx Plus, STARR Life Sciences Corp.), using a rectal probe feedback controlled heating pad (TC-1000 Temperature Controller, CWE Inc.). Two multi-electrode arrays (MEA, MicroProbes, Gaithersburg, MD, United States) comprising 16 Platinum/Iridium electrodes (organized in a grid of evenly spaced 4 rows and 4 columns, tip diameter: 125 μm, impedance: 0.5 MΩ, inter-electrode spacing: 250 μm, for a total MEA width of 1 mm and a total length of 1 mm) were lowered to 250 μm DV into the exposed cortices for intracortical recordings. The MEA Centre was positioned at -1.0 mm AP and -2.5 mm ML to allow for placement of the first row of electrodes ∼0.5 mm posterior to the caudal injection site. Acquisition bandwidth was set to 0.3–5 kHz. Signals were amplified 20× at the head-stage and 50× by the amplifier (Model 3600, A-M Systems, Carlsborg, WA, United States). Data were acquired using a 32-channel SciWorks DataWave Acquisition System, with a sampling rate set to 20 kHz, and stored for offline analysis.

### Electrophysiological Data Analysis

Power line noise (60 Hz) and its harmonics were removed via notch filtering before additional filtering using a zero-phase forward and reverse Butterworth infinite impulse response filter with frequency range of ±2 Hz around the stop band (filtfilt.m in Matlab, MathWorks, Natick, MA, United States) to eliminate phase distortions (McGinn and Valiante, 2014). To estimate power, the average signal of each row of four electrodes was computed and then Fast Fourier Transform computed for all 3-s intervals in each 3-min epoch using a non-overlapping running window. The relative power was calculated as the fraction of a specific frequency band power vs. the total power over all frequency bands. Following previous work (Joo et al., 2017; Bazzigaluppi et al., 2018), the frequency bands of interest were defined as: Theta (2–8 Hz), Alpha (10–14 Hz), Beta (15–30 Hz), low Gamma (Laird and Ware, 1982; Bragin et al., 1995; Roberts et al., 1996; Simpkins et al., 1997; Chrobak and Buzsaki, 1998; Hendrich et al., 2001; Guilfoyle et al., 2003; Kang et al., 2003; Lee et al., 2005; Miller et al., 2005; Canolty et al., 2006; Takuma et al., 2007a,b; Hossmann, 2008, 2009; Dudink et al., 2009; Canolty and Knight, 2010; de Hemptinne et al., 2013; Girard et al., 2014; McGinn and Valiante, 2014; Womelsdorf et al., 2014; Lake et al., 2015; Edakawa et al., 2016; Zhang et al., 2016; Barr et al., 2017; Devergnas et al., 2017; Gomez-Smith et al., 2017; Joo et al., 2017; Bazzigaluppi et al., 2018) and high Gamma (62–120 Hz). Power exceeding 2 Median Absolute Deviations from band’s median (estimated independently per surgery group, ROI, and contrast) were excluded from further analysis. Neuronal dynamic motifs present in the cortex integrate synaptic input and specify spike output synchronization and have been hypothesized to give rise to these frequency bands (Womelsdorf et al., 2014). The functional interactions between frequency bands have been observed in both animals and humans and are termed cross-frequency coupling [CFC, (Canolty and Knight, 2010) and references therein]. CFC focuses on the properties of the ongoing oscillatory activity itself and reflects the statistical dependence between distinct frequency bands of the ongoing spontaneous electrical activity rather than dependence of the electrical activity on external stimuli. One of the manifestations of CFC is Phase Amplitude Coupling (PAC), where the phase of a low frequency wave (theta) modulates the amplitude of higher frequency band [low and high gamma, (Bragin et al., 1995; Chrobak and Buzsaki, 1998; Lee et al., 2005)]. In the present work, we investigated the phase-amplitude coupling by estimating the Modulation Index (MI) that combines the amplitude envelope of time series (A) of a high-frequency band (low and high gamma, A_gamma_) with the phase φ(t) of a low-frequency band (theta, φ_theta_) into one composite signal z(t). We estimated the temporal profile (i.e., time series) of the joint distribution of A_gamma_ and φ_theta_. To achieve this, following earlier work (Canolty et al., 2006; Bazzigaluppi et al., 2018), we compared the mean of the amplitude of z(t) signal to a set of surrogate means created by offsetting A_gamma_ and φ_theta_ by a large time lag (to create random distributions of amplitudes and phases). The time lag affects only the dependence between A_gamma_ and φ_theta_, not the distributions of amplitude and phase themselves. We used a set of 50 surrogates to generate a population of spurious MI used to threshold (after Bonferroni correction for multiple comparisons), at an alpha of 0.05, the MI extracted from the raw dataset. The mean over time of this composite signal (the Modulation Index) represents the strength of coupling between the two frequency bands. Raw recordings were re-referenced offline between two neighboring recording sites and modulation index was estimated with an in-house developed Matlab function. Theta band was divided into 0.2 Hz bins, while both gamma bands were binned every 1 Hz. Following previous work (Lake et al., 2017), results of neuronal power and MI population analyses are expressed as *lateralization* (i.e., the ratio of the peri-to contra-lesional values) to evaluate the deficit of the peri-lesional tissue in relation to the notionally unaffected contralesional hemisphere.

### ELISA

Whole blood samples were collected at the beginning of ovariectomy or sham surgery, at the beginning of ET-1 stroke induction surgery, and at the start of MRI sessions. Following clean venipuncture, we collected 300 to 500 μL of whole blood from the lateral tail vein immediately after induction with anesthesia. Samples were kept at 4°C for approximately 45 min to facilitate coagulation. Serum was isolated after centrifugation (21K rpm, 15 min, 4 C), then frozen at -20°C as individual aliquots for each rat and at each time point (serum was not pooled). Each sample of serum was divided into three 25 μL aliquot and assayed for Estradiol (Calbiotech Estradiol Elisa Kit, #ES180S-100) as per manufacturer’s instructions. The estradiol reaction was measured at 450 nm absorbance and relative fluorescence fitted to the reference standard curve.

### Statistical Analysis

Unless stated otherwise, linear mixed effects (lme) modeling (lme function in R^1^) was used in the statistical analysis. It is particularly well suited for the present data since it produces robust and sensible maximum likelihood estimates in the presence of unbalanced number of subjects in experimental groups, here present due to attrition rates variation (Laird and Ware, 1982). CASL-based estimates of resting perfusion and cerebrovascular reactivity to hypercapnia as well as intracortical electrophysiological recordings-based measurement of spontaneous neuronal activity power and modulation index estimates were modeled as linear functions of the surgery (Sham or Ovx), which is the fixed effect, while subjects were treated as random effects. Results are expressed as mean ± SEM unless specified otherwise.

## Results

**FIGURE 1:**
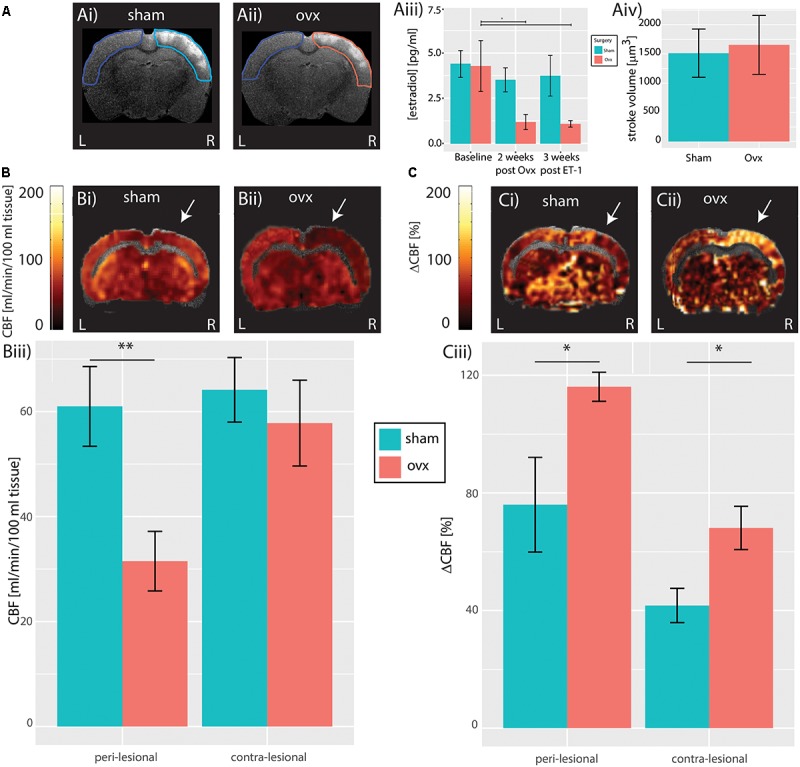
Cerebral hemodynamics. **(A)** Structural T_2_-weighted MR images of a Sham **(Ai)** and Ovx subject **(Aii)** showing anatomically identified peri-lesional tissue in turquoise and pink, respectively, and contra-lesional tissue in blue used for estimation of stroke volume; population analysis of T_2_-weighted hyperintensity does not reveal difference in stroke volume between Sham and Ovx subjects **(Aiii)**. **(B)** Resting perfusion in a representative Sham **(Bi)** and Ovx **(Bii)** animal. Population analysis revealed reduced blood-flow in the peri-lesional (but not in the contra-lesional) tissue of Ovx subjects compared to that of the Sham group **(Biii)**. White arrow indicate the region of the slice most proximal to ET-1 injection sites. **(C)** Response to hypercapnia in a representative Sham **(Ci)** and Ovx **(Cii)** animals. Population analysis revealed increased response to hypercapnia both in the peri-lesional and in the contra-lesional tissue of Ovx animals compared to the Sham group **(Ciii)**. ^∗^*p* < 0.05 and ^∗∗^*p* < 0.01.

We employed the endothelin-1 (ET-1) model (Lake et al., 2017) to induce focal ischemia in the right sensorimotor cortex of Sham and Ovx female rats and measured the spontaneous neurovascular recovery in the chronic stage. To model the reduction in circulating estradiol following oophorectomy, we performed Ovx or Sham surgery. When compared to baseline (i.e., before Ovx or Sham surgery), 2 weeks post Ovx (i.e., just before stroke induction), Ovx rats exhibited a 68% drop in blood estradiol concentration (4.3 ± 1.4 pg/ml vs. 1.2 ± 0.4 pg/ml, *p* = 0.024) as shown in **Figure 1Aiii**, whereas Sham animals did not show a significant difference (4.4 ± 1.6 pg/ml vs. 3.5 ± 0.7 pg/ml, *p* > 0.05). Three weeks after ET-1 induction, blood estradiol concentration settled at 1.1 ± 0.2 pg/ml (*n* = 3, *p* = 0.6, vs. baseline) in Ovx animals and at 3.8 ± 1.1 pg/ml (*n* = 2, *p* = 0.1 vs. baseline) in Sham animals. At the study end point, Ovx rats did not show a significant drop in body weight when compared to Sham (414 ± 23 g vs. 475 ± 54 g, *p* = 0.23).

### Peri-Lesional Hypoperfusion and Bilateral Hyperreactivity of Ovx Females

To assess the effects of ovariectomy on stroke volume post ischemic insult, *in vivo* T_2_-weighted MR-images were acquired in all animals 3 weeks after insult. Stroke volumes in both cohorts were estimated by segmenting regions of signal hyperintensity in the peri-lesional tissue (Dudink et al., 2009). Ovariectomy did not affect peri-lesional tissue volume, (Ovx: 1654.7 ± 504.6 mm^3^, *n* = 6 vs. Sham: 1513.1 ± 414.0 mm^3^, *n* = 6, *p* = 0.98, Student’s *t*-test, **Figure 1Aiv**). Given the limited sensitivity of T_2_-weighted MRI in predicting vascular and functional recovery [(Girard et al., 2014) and for review (Lake et al., 2016)], CASL MRI data were collected and used to estimate resting brain perfusion and cerebrovascular reactivity to hypercapnia, a standard (pre)clinical test of brain vascular functioning. CASL maps overlaid on structural T_2_-weighted MR images from representative Sham and Ovx animals 3 weeks after surgery are shown in **Figure 1** (resting perfusion in **Figure 1B** and cerebrovascular reactivity to hypercapnia in **Figure 1C**). When compared to Sham animals, Ovx rats showed reduced resting perfusion in the peri-lesional cortex (60.9 ± 7.6 ml/min/100 g, *n* = 6, vs. 31.5 ± 5.7 ml/min/100 g, *n* = 6, *p* = 0.0026, Student’s *t*-test), but not in the contralateral cortex (64.1 ± 6.1 ml/min/100 g, *n* = 6, vs. 57.8 ± 8.2 ml/min/100 g, *n* = 6, *p* = 0.2975, Student’s *t*-test), as summarized in **Figure 1Biii**. The lateralization (i.e., the peri-to-contralesional ratio of resting perfusion) was significantly below 1 in the Ovx cohort (*p* = 0.0016), but indistinguishable from 1 in the Sham cohort (*p* = 0.25). Ovx animals showed increased cerebrovascular reactivity to hypercapnia (**Figure 1C**) when compared to Sham animals both in the peri-lesional cortex (76 ± 16.1%, *n* = 6, Sham vs. 116.2 ± 4.9%, *n* = 6, Ovx, *p* = 0.0384, Student’s *t*-test), and in the contra-lateral cortex (41.7 ± 5.8%, *n* = 6, Sham vs. 68.1 ± 7.3%, *n* = 6, Ovx, *p* = 0.0254, Student’s *t*-test), indicating exacerbated cerebrovascular dysfunction in Ovx relative to Sham animals. The lateralization of the cerebrovascular response to hypercapnia was evident both in the Ovx (*n* = 6, *p* = 6.3^∗^10^-5^) and in the Sham cohort (*n* = 6, *p* = 0.0055), indicating sustained hyper-reactivity of the peri-lesional vasculature in both cohorts.

### Preserved Neuronal Power in Ovx Females

**FIGURE 2:**
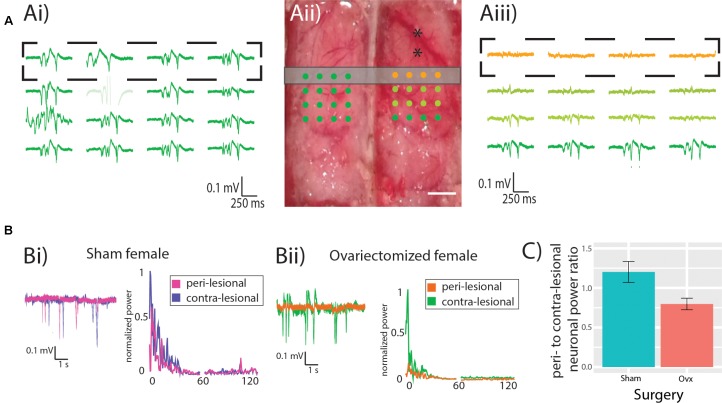
Neuronal power. **(A)** Representative recording from contra- **(Ai)** and ipsi-lesional **(Aiii)** hemispheres, showing in orange the recordings from the peri-lesional tissue. Experimental design showing the MEA location (dots) in relation to the ET-1 injection sites (black asterisks) and the location of the CASL imaging slice (gray rectangle). Scale bar 0.5 mm. **(B)** representative recordings and power spectrum from the peri-lesional and contra-lesional hemisphere of Sham **(Bi)** and Ovx **(Bii)** animal. **(C)** Ovx animals (red bar) show a trend toward reduced neuronal power in the peri-lesional tissue when compared to that of Sham animals (green bar).

**Figure 2A** shows multi-electrode array recordings from the contralesional hemisphere (**Figure 2Ai**) and from the peri-lesional hemisphere (**Figure 2Aiii**) of an Ovx female (electrode arrays positioning is shown in **Figure 2Aii**). The spontaneous neuronal activity in the peri-ischemic area (orange traces in **Figure 2Aiii**) was attenuated in comparison to that in the caudal area of the ipsilesional hemisphere and to that of the contralesional hemisphere (green traces in **Figures 2Aii,iii**). **Figure 2Bi** displays example traces from the peri-ischemic tissue of a Sham female (magenta trace in **Figure 2Bi**) and the recording from the homologous electrode in the contra-lesional hemisphere (purple trace in **Figure 2Bi**) and their power spectra. Example traces in an Ovx female are enlarged in **Figure 2Bii** (orange for the peri-ischemic and green for the contralesional) alongside their power spectra. **Figure 2C** shows the lateralization of total neuronal power in the Ovx vs. Sham females, revealing a trend toward greater peri- vs. contra-lesional neuronal power attenuation in the Ovx cohort when compared to the Sham group (1.2 ± 0.1, *n* = 6, vs. 0.8 ± 0.1, *n* = 6, *p* = 0.07). There were no differences in the ratio of relative neuronal power of Ovx vs. Sham animals in any of the individual bands (Theta: 0.71 ± 0.17 vs. 0.41 ± 0.05, *p* = 0.1; Alpha: 0.98 ± 0.3 vs. 0.68 ± 0.8, *p* = 0.41; Beta: 0.99 ± 0.2 vs. 0.73 ± 0.07, *p* = 0.28; Low gamma: 1.4 ± 0.2 vs. 0.96 ± 0.14, *p* = 0.1; High gamma: 1.92 ± 0.35 vs. 1.21 ± 0.18, *p* = 0.1, Student’s *t*-test). The trend toward decreased power ratio observed in the peri-lesional tissue of Ovx rats was lost at increasing distances from the ET-1 injection site, namely at 0.5, 1, and 1.5 mm from the peri-lesional tissue, indicating that the neuronal dysfunction was spatially restricted.

### Reduced Theta to Low Gamma Phase Amplitude Coupling in Ovx Females

**FIGURE 3:**
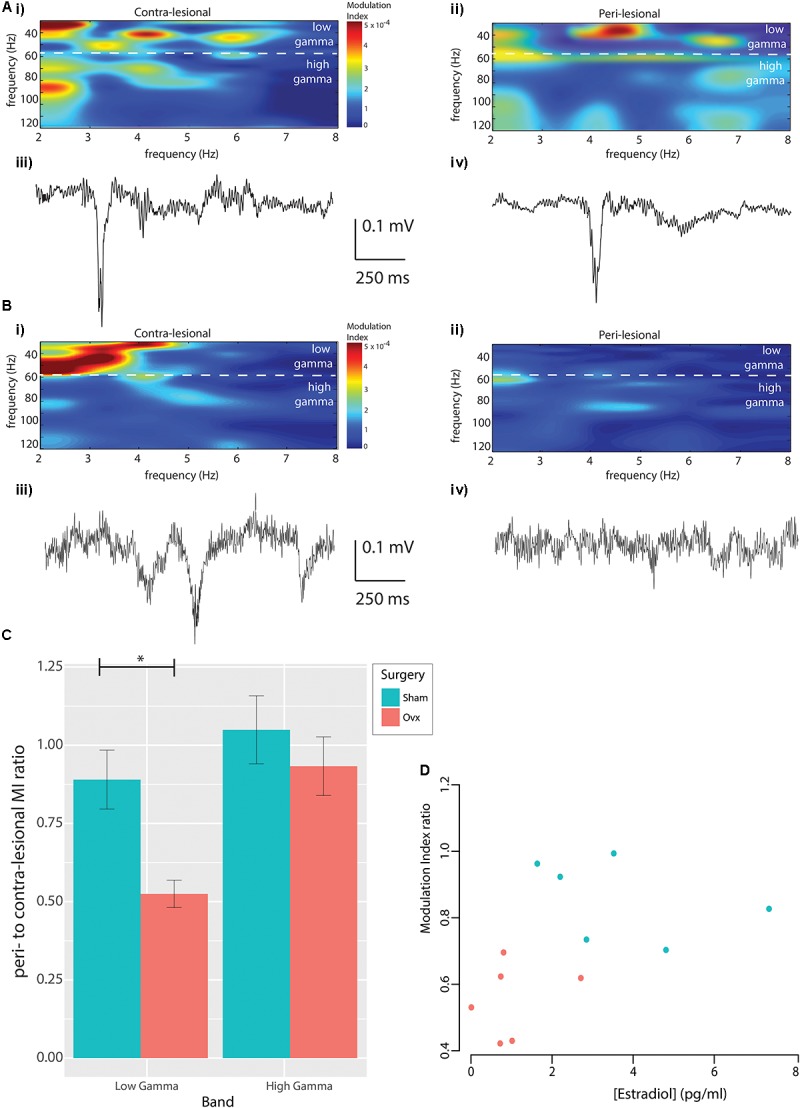
Modulation index. **(A)** Modulation Index from contra-lesional **(Ai)** and peri-lesional **(Aii)** regions of a representative Sham rat, with representative raw data epochs from contra-lesional **(Aiii)** and ipsi-lesional **(Aiv)** regions of the same rat. **(B)** The corresponding data in an Ovx animal. **(C)** Population analysis revealed reduced peri-to-contralesional theta to low-gamma MI ratio in the Ovx vs. Sham cohort, whereas theta to high-gamma MI ratios were indistinguishable. **(D)** Positive correlation between peri-to-contra-lesional theta to low-gamma MI ratio and serum estradiol concentration at stroke-induction (Spearman’s correlation, rho = 0.67, *p* = 0.04).

In light of the trends observed in neuronal power and the sensitivity of Cross Frequency Coupling in capturing neuronal dysfunction in pathological models even in the absence of changes in power (de Hemptinne et al., 2013; Edakawa et al., 2016; Zhang et al., 2016; Barr et al., 2017; Devergnas et al., 2017; Bazzigaluppi et al., 2018), we explored Cross Frequency Coupling (CFC) between theta and gamma bands. Contra- and ipsi-lesional MI and sample recordings from a representative Sham animal are displayed in **Figure 3A**, while corresponding data from an Ovx animal are presented in **Figure 3B**. Representative data from the whole MEA are presented in **Supplementary Figure S1**. Population analysis revealed that the average theta to low-gamma MI was significantly reduced in Ovx animals (**Figure 3C**, 0.89 ± 0.10 Sham, *n* = 6, vs. 0.52 ± 0.04 Ovx, *n* = 6, *p* = 0.009, Student’s *t*-test), while theta to high-gamma MI was not affected (**Figure 3C**, 1.04 ± 0.1 Sham, *n* = 6, vs. 0.93 ± 0.1 Ovx, *n* = 6, *p* = 0.42, Student’s *t*-test). Correlating the lateralization in theta to low-gamma MI with the estradiol concentration at the time of ET-1 injection revealed a positive correlation between the two variables (**Figure 3D**, Spearman’s rank correlation coefficient rho = 0.67, *p* = 0.04): higher estradiol levels correlated with reduced MI lateralization in the chronic stage. Furthermore, the use of MEA allowed us to describe the topology of the lesion: theta to low-gamma MI lateralization in Sham and Ovx declined at increasing distances from the peri-lesional tissue (tau = -0.7, Spearman’s rank correlation, *p* = 0.08; theta to high gamma: tau = 0, Kendall’s rank correlation, *p* = 1). The deficit in functional interaction between neuronal ensembles in the chronic stage of injury was thus confined to the tissue within 0.5 mm AP from the injection site.

## Discussion

It is known from clinical studies that bilateral oophorectomy in premenopausal women and the consequent drop in ovarian hormonal levels are associated with increased multimorbidity with 18 defined chronic conditions (Levine et al., 2016; Rocca et al., 2017). Furthermore, women approaching menopausal age are subject to an increased risk of stroke and reduced recovery compared to age matched men (Anderson et al., 1991). In the attempt to prevent the pernicious effects of hormonal depletion observed in aging women, estrogen replacement therapy (ERT) has been sporadically attempted as of 1960s. However, following experimental data from the Women’s Health Initiative (WHI) and the observational data from the Nurses’ Health Study (NHS) (Biernaskie et al., 2001) showing increased risk for secondary stroke in women older than 50 years supplemented orally with estrogen, it was considered an invariable risk factor for stroke and gradually abandoned. New studies [(Biernaskie et al., 2001) and references therein] proposed and supported the notion of a *timing hypothesis*, which posits that ERT exerts beneficial effects in patients younger than 50 years and starts becoming detrimental for women older than 60, particularly if administered orally. Testing of this hypothesis and the design of novel therapeutic approaches based on hormonal (estrogen alone or in combination with progesterone or allopregnanolone) supplementation requires models that recapitulate the salient phases of focal cerebrovascular occlusion-reperfusion and the drop in ovarian hormones observed in the patient population following bilateral oophorectomy. The present study is the first to link *in vivo* neurovascular function, which has been demonstrated a sensitive assay of brain recovery [for review (Lake et al., 2016)], with circulating estrogen levels at the time of ischemia, providing initial evidence that the evolution of ischemic damage and recovery is affected by estrogen levels in a model of oophorectomy in mature females. Our model of oophorectomy reproduces the faster decrease in estrogen concentration observed in surgically induced menopause [vs. that of natural menopause in women (Korse et al., 2009)] and ET-1 model of focal cortical ischemia produces a precisely targeted necrotic core surrounded by a sizeable region of functionally-challenged peri-ischemic tissue (Hossmann, 2008, 2009). Of note, in our previous study using this stroke model in males, we observed a mortality of ∼12% (Lake et al., 2017) at 48 h post-stroke; in the current study, mortality following stroke injection was 25% in Sham (2 in 8) and 40% in Ovx (4 in 10) females at the same time point (*p* = 0.52 Chi Squared test), in line with previously reported 35 and 54% of mortality at 6 and 24 h, respectively, in Charles River rats following transient Middle Cerebral Artery occlusion (Simpkins et al., 1997). Although mortality was not significantly different between groups at present, larger cohort studies may reveal the survival as a useful outcome measure of the model.

### Neuronal Dysfunction

It was shown by Takuma and collaborators in female Fisher rats that Ovx alone (as well as in combination with restraint-stress) causes pyramidal neurons death in the CA3 and Dentate Gyrus of the hippocampus, associated with cognitive deficits in the novel object recognition test (Takuma et al., 2007a,b). Furthermore, it was previously reported that normo-oestrus female rats have lower death rate (Simpkins et al., 1997) and reduced neuronal death in the hippocampus (Miller et al., 2005; Zhang et al., 2006) and cortex (Fukuda et al., 2000) following global ischemia when compared to estrogen-depleted rats [for review (Brann et al., 2007)]. However, the effect of estrogen depletion/supplementation have mainly been investigated in the early state and by post-mortem assays, which cannot describe functional neuronal recovery (McCullough and Hurn, 2003). In the sole report describing the effect of Ovx on neuronal function in the acute stage of ischemia, Pelligrino et al. (1998) observed larger reduction in neuronal power within the first 30 min following common carotid artery clamping combined with hemorrhagic hypotension in Ovx female rats compared to Sham animals. However, early neuronal dysfunction is not a reliable predictor of long term injury (Moore et al., 2000; Lake et al., 2017; Momosaki et al., 2017). To develop novel therapeutic approaches, it is necessary to have a model that recapitulates the combined effect of estrogen loss and the sequelae of events following ischemia. The present study provides novel data on the consequences of estrogen depletion on neuronal and cerebrovascular recovery in the chronic stage, at 3 weeks post-stroke. Increased excitability in the peri-lesional cortex has been observed in male rats 3 weeks post stroke by us (Lake et al., 2017) and between a week to a month post stroke by others (Schiene et al., 1996; Winship and Murphy, 2008). The role of this increased excitability is still a matter of debate, but it has been hypothesized to be necessary for rewiring of the neuronal network [for review (Rabiller et al., 2015)]. In the present work, we did not observe increased neuronal power in the peri-lesional tissue of either Sham or Ovx females. In the absence of such dynamics in the females, and in the absence of a strong link between neuronal survival on histopathological assays and long-term functioning (Corbett and Nurse, 1998), we focused on the coupling between the phase of slow oscillation (theta) and the amplitude of a faster oscillation (gamma) since PAC has been hypothesized to be a sensitive predictor of cognitive function (Tort et al., 2009; Canolty and Knight, 2010; Lisman and Jensen, 2013). Although the features of excitatory and inhibitory network connectivity underlying PAC have not been established, PAC is known to reflect the temporal coordination of neuronal networks across or within brain regions (Womelsdorf et al., 2014) and has been observed in humans (Palva et al., 2005; Canolty et al., 2006) and rodents [for review (Jensen and Colgin, 2007)]. We focused on the modulation that the theta band (2–8 Hz) exerts on low-gamma band (30–60 Hz) and high-gamma band (60–120 Hz), as its importance has been shown in recent studies of human neocortical injury (Canolty et al., 2006; Jensen and Colgin, 2007). Patients with schizophrenia, Autism Spectrum Disorder, and Parkinson’s Disease show reduced phase locking of gamma-band oscillations to theta and beta bands (Uhlhaas and Singer, 2012; Oswal et al., 2013), Mild Cognitive Impairment (Dimitriadis et al., 2015) and Alzheimer’s Disease patients, in turn, exhibit reduced coupling between the phase of theta and the amplitude of gamma bands (Poza et al., 2017). Our results show for the first time that in the chronic stage, peri-lesional tissue of Ovx animals presents a ∼40% reduction in theta to low-gamma (but not theta to high-gamma) modulation when compared to that seen in Sham rats. While the difference between low gamma and high gamma bands is still unclear (Ray and Maunsell, 2011), they are known to be modulated independently. In humans, low gamma is mainly modulated by attention (Roh et al., 2016), while high gamma activity is modulated by sensory, motor, and cognitive events (Crone et al., 1998; Edwards et al., 2005). Moreover, the amplitude of both bands, is modulated (though in opposing directions) in relation to the gait cycle (Seeber et al., 2015). Reduced temporo-parietal PAC has been shown to be associated with dementia and Alzheimer’s Disease in patients (Poza et al., 2017), while an increase in subtalamic beta to gamma PAC is associated with increased severity of motor symptoms in Parkinson patients (van Wijk et al., 2016). Moreover, estradiol concentration measured at the time of stroke induction predicted the extent of this deficit, with higher estradiol concentration correlating with less injury (reduced lateralization of the modulation index). This observation lends further support to the importance of estradiol in injury susceptibility/functional recovery.

### Cerebrovascular Dysfunction

It was previously reported that estrogen deficiency does not affect resting CBF measured by photoelectric method in urethane or halothane anesthetized animals (Holschneider and Scremin, 1998; Szelke et al., 2008), which have both been shown to depress cortical blood flow (Ueki et al., 1992). Propofol anesthesia, used presently, mildly reduces CBF, preserving normal circulation and metabolism (Oshima et al., 2002). Our experiments show that in the chronic state following ischemic insult, Ovx animals show ∼50% reduction in resting perfusion in the peri-lesional tissue, but not in the contralateral cortex. While we cannot exclude that CBF was reduced at the time of ET-1 injection in Ovx animals, **Figure 1Bii** shows that the resting perfusion in the contralesional (i.e., notionally unaffected hemisphere) 3 weeks after stroke induction was not distinguishable between Ovx and Sham animals. Notwithstanding, further studies on the effects of altered estradiol levels on resting cerebral perfusion are required. Furthermore, the resting CBF is lateralized (attenuated peri-lesionally vs. contra-laterally) in the Ovx but not in the Sham cohort. We have previously reported in the same ET-1 stroke model in adult male rats that resting cerebral blood flow normalizes in the chronic stage post stroke (Lake et al., 2017), just as it did here in the Sham females. However, in the same report we showed that neuronal power was elevated 3 weeks post stroke, supporting the notion of sex-differences in neurovascular recovery [and development in general (Manwani and McCullough, 2011)]. Previous work in the hyperacute stage of stroke showed that Ovx exacerbated CBF drop (measured with laser-Doppler flowmetry LDF) in the first half-hour following common carotid artery clamping combined with hemorrhagic hypotension (Pelligrino et al., 1998), and the increased drop be mediated by reduced nitric oxide synthase activity [for review (Pelligrino and Galea, 2001)]. In the present work we report that in response to hypercapnia, Ovx animals showed a ∼50% increase in cerebrovascular reactivity when compared to Sham animals both peri- and contra-lesionally. Previously it was reported that cerebrovascular responsivity to hypercapnia increases of ∼14% in the absence of cortical damage measured with the photoelectric method (Szelke et al., 2008). Flow response to hypercapnia relies on arteriolar smooth muscle relaxation (Mellander, 1989) and previous *ex vivo* report using the Pressurized Arteriograph Chamber showed that 30 days following Ovx, myogenic reactivity and passive lumen diameter of penetrating arterioles were unaffected while myogenic tone and Blood–Brain Barrier (BBB) permeability to Lucifer Yellow were increased when compared to specimens harvested from control subjects (Cipolla et al., 2009). In a different study, *ex vivo* spectrophotometry revealed increased BBB permeability to Evans Blue following global ischemia-reperfusion in Ovx female rats 3 weeks following surgery (Uzum et al., 2015). In both cases, ERT rescued the increase in BBB permeability. This evidence, together with the observation that BBB is more permeable in reproductively senescent females in the absence of stroke (Bake and Sohrabji, 2004) or acutely after experimental stoke (DiNapoli et al., 2008), indicates that the chronic stage neurovascular damage may be associated with increased BBB permeability.

### Study Shortcomings and Translational Potential

As mentioned earlier, epidemiological studies have demonstrated that postmenopausal women have a higher risk of stroke than do age-matched men. While hypertension, diabetes, smoking and obesity are frequent, *modifiable* risk factors in both sexes, biological sex, aging and menopause are *non-modifiable* risk factors. To date, therapeutic exploitation of perimenopausal hormonal influence on cellular and molecular mechanisms underlying neuronal deficits following ischemia has been hindered by the lack of establishment of the neurovascular loss of function in the chronic stage observed in the patient population (Koellhoffer and McCullough, 2013). The present work provides a model that recapitulates the ovariectomy-induced exacerbation of peri-lesional cerebrovascular and neuronal dysfunction, which was found correlated with the circulating estradiol level at the time of stroke induction. PAC is still underutilized as recovery predictor in the clinic; rather, delta and theta bands power have been suggested highly sensitive markers of hypoxia [for review (Rabiller et al., 2015)]. Cerebral blood flow and cerebrovascular reactivity (CVR) mapping provides valuable information in the evaluation of cerebrovascular injury, including arterial stenosis, stroke, small vessel disease, brain tumors, traumatic brain injury, and normal aging [for review (Liu et al., 2017)]. Furthermore, both CBF and CVR can be measured non-invasively and are readily accessible in the patient population (Liu et al., 2017). While a battery of sensitive behavioral tests of sensorimotor function will be necessary to assess the benefits of any therapeutic interventions, the present findings reveal significant effects of ovariectomy on translationally relevant markers of neurovascular recovery from stroke.

## Author Contributions

PB, AP, PC,and BS designed the study and wrote the manuscript. PB, CA, and MK performed the experiments.

## Conflict of Interest Statement

The authors declare that the research was conducted in the absence of any commercial or financial relationships that could be construed as a potential conflict of interest.

## Footnotes

1 http://www.R-project.org
