# Supplementary material for: Oophorectomy Reduces Estradiol Levels and Long-Term Spontaneous Neurovascular Recovery in a Female Rat Model of Focal Ischemic Stroke
Source: Front Mol Neurosci. 2018 Sep 13;11:338. doi: 10.3389/fnmol.2018.00338 (PMC6146137; doi:10.3389/fnmol.2018.00338)
Supplement: Supplementary file 1 [file Data_Sheet_1.docx]

***EPI readout details:***

Echo position: 29.8%, EPI ramp time: 0.08ms, target gradient slope: 80%, Blip duration: 0.1m, Echo delay: 6.5ms, EPI module duration: 19.9ms, gradient shape dwell time: 10ms Echo spacing 0.320ms, effective image bandwidth: 3125Hz, dephasing time: 0.742ms, dephasing ramp time: 0.131ms, plateau duration: 0.160ms, acquisition delay: 0.82ms, inter-ramp time: 0.08ms, gradient switching time: 0.16ms, readout dephasing: -0.0862, readout gradient odd: -0.4392, readout gradient even: 0.4392, phase dephasing: -0.048, phase rephrasing: -0.113, blip amplitude odd/even: 0.03514, Echo train length: 57, Echo counter: 28, PVM_EpiRampIntegrals: 0.5, gradient slew rate: 3667.84 T/m/sec, number of samples per scan: 4560, number of scans: 584; Ghost correction was on, with the following parameters: Acquisition delay correction: 18 ms, dephasing correction 100%, and Grappa SVD threshold: 0.05.

***Four saturation slices were used (supplementary figure 1C), with the following pulse parameters***:

Length: 1ms, Bandwidth: 5400Hz, flip angle: 90 degrees, attenuation: 3.4 dB, trim bandwidth: 100%, trim attenuation: 0%, trim rephase: 100%, bandwidth factor: 5400 Hz*s, integral ratio: 0.1794, rephrase factor: 50%, minimum pulse length: 0.102ms.

***Supplementary figure 1: MR imaging***

In A: position of the imaging coil (Ai) relative to the labelling coil was then determined with a localizer acquired using the labelling coil (Aii) to ensure optimal labelling offset in all subjects; in B: Fastmap sequence was run to adjust shims in the longitudinal (Bi) and coronal (Bii) planes in the brain region to be imaged; in C: saturation slices (in purple) on the sides of the region of the brain to be imaged in the longitudinal (Ci) and coronal (Cii) planes.

***Supplementary figure 2: Topology of theta to low gamma MI lateralization.***

Raw recordings were re-referenced offline between two neighboring recording sites to estimate phase-amplitude coupling (see methods for details). Theta to low-gamma MI from a representative Ovx animal are shown in figure Ai (contra-lesional hemisphere) and in figure Aii (peri-lesional hemisphere), showing reduced theta to low-gamma MI. For comparison, theta to low-gamma MI from a representative Sham animal are shown in figure Bi (contra-lesional hemisphere) and in figure Bii (peri-lesional hemisphere).
