# Supplementary figures and images for: Oophorectomy Reduces Estradiol Levels and Long-Term Spontaneous Neurovascular Recovery in a Female Rat Model of Focal Ischemic Stroke
Source: Front Mol Neurosci. 2018 Sep 13;11:338. doi: 10.3389/fnmol.2018.00338 (PMC6146137; doi:10.3389/fnmol.2018.00338)

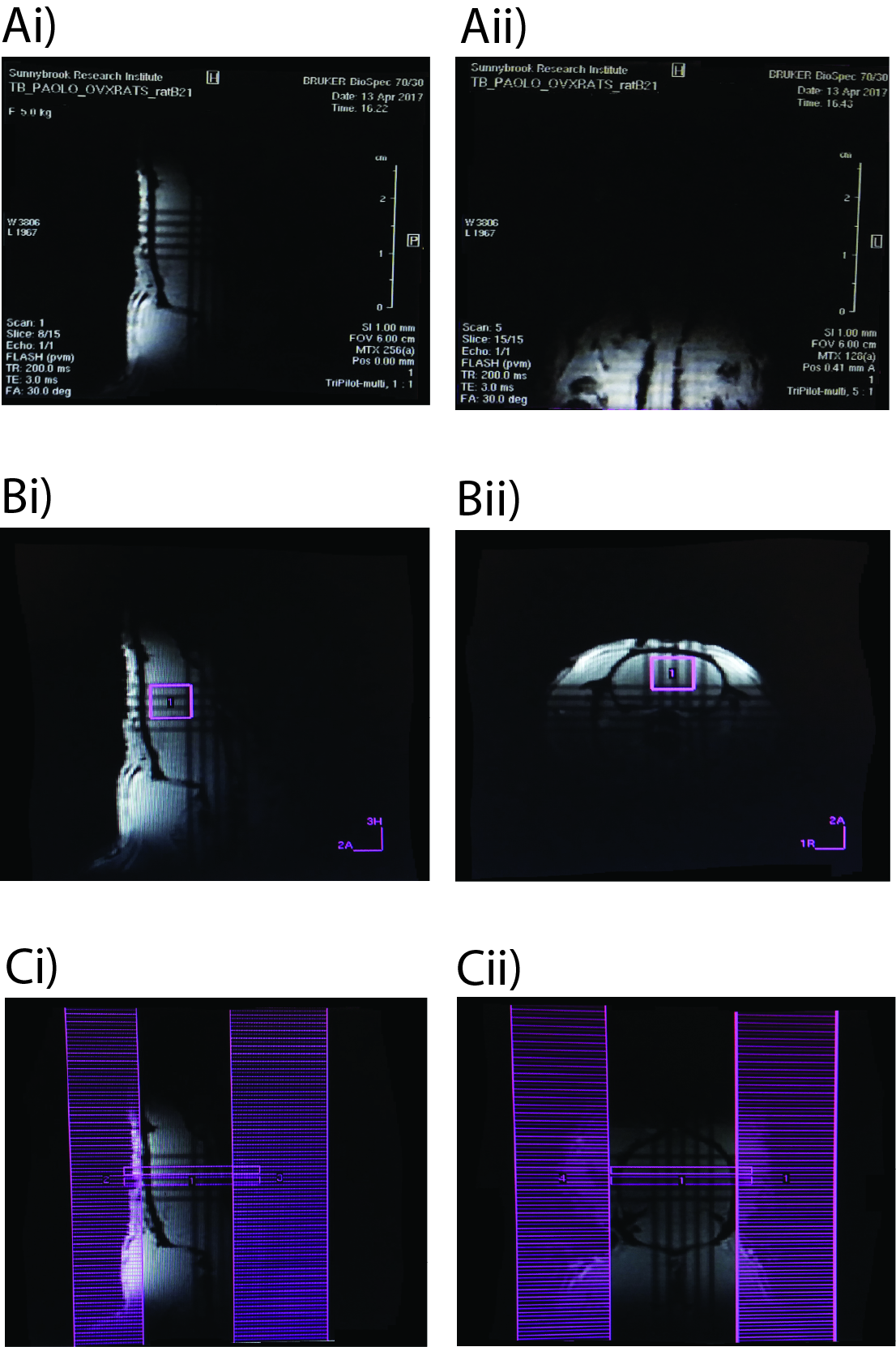

Supplement: Supplementary file 2 [file Image_1.TIF]

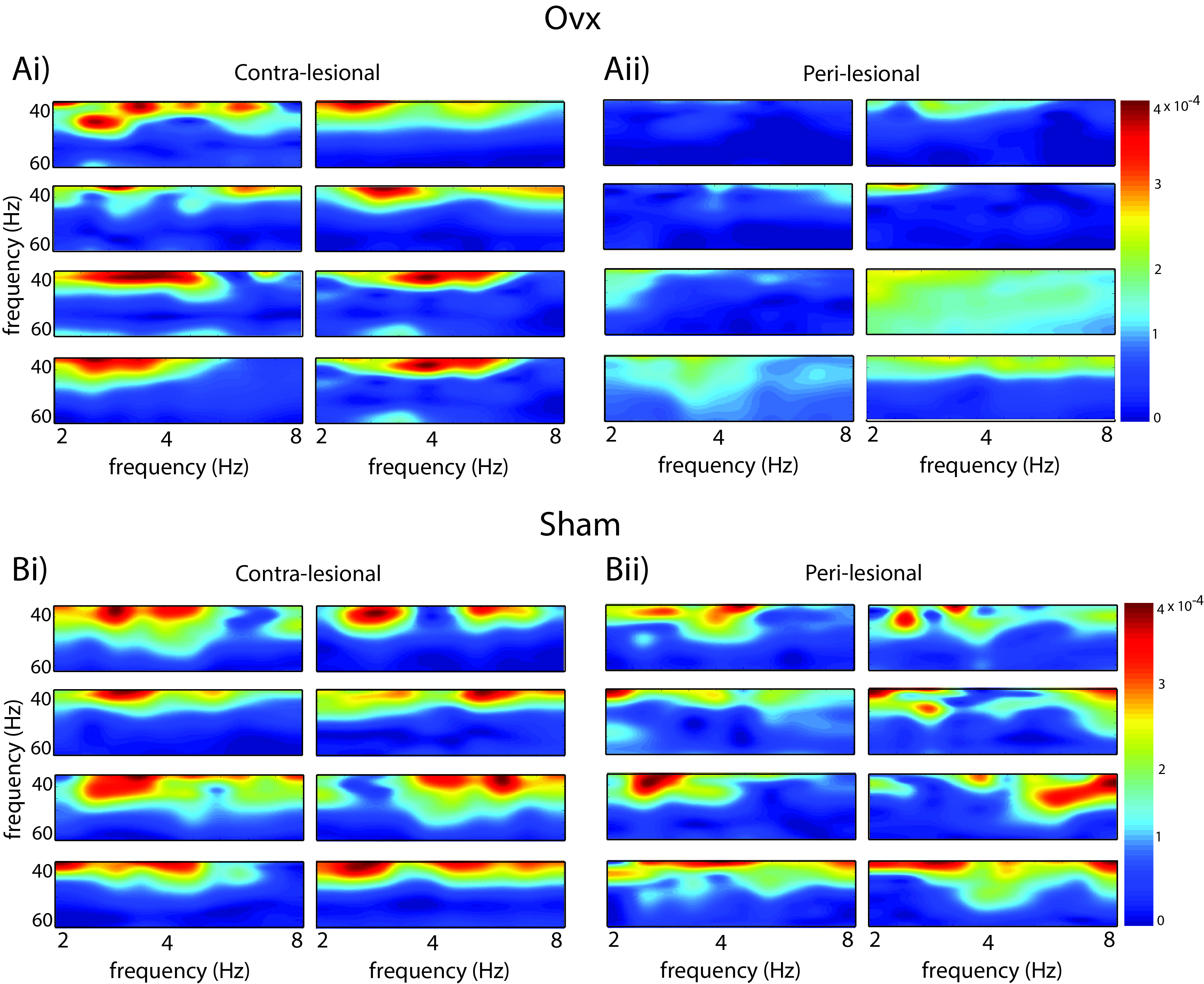

Supplement: Supplementary file 3 [file Image_2.TIF]
